# Supplementary material for: The Effects of 1-Deoxynojirimycin from Mulberry on Oxidative Stress and Inflammation in Laying Hens and the Direct Effects on Intestine Epithelium Cells In Vitro
Source: Animals (Basel). 2023 Sep 6;13(18):2830. doi: 10.3390/ani13182830 (PMC10525092; doi:10.3390/ani13182830)
Supplement: Supplementary file 1 [file animals-13-02830-s001.zip › animals-2531126-supplementary.pdf]

Supplementary Materials: Tables

Table S1 Ingredients and nutrient levels of experiment diet

Item	%	Nutrient level[b]	level
Corn	65.50	Metabolic energy (MJ/kg)	11.45
Soybean meal	24.00	Crude Protein, %	16.13
Shell powder	5.80	Calcium, %	3.32
Limestone powder	1.78	Available Phosphorus, %	0.33
Calcium hydrogen phosphate	1.30	Methionine, %	0.33
NaCl	0.30	Lysine, %	0.96
50％ choline chloride	0.20		
Methionine	0.12		
Premix[a]	1.00		
Total	100.00		

[a] Premix provided per kilogram of diets: vitamin A ((retinyl palmitate) 7 715 IU; vitamin D_3 2 755 IU， vitamin E 8.8 IU, vitamin 2.2 mg, vitamin B_{12} 0.01 mg, vitamin B_2 4.41 mg, vitamin B_3 5.51 mg, vitamin B 0.55 mg， niacin 19.8 mg, folic acid 0.28 mg, Mn 50 mg, Fe 25 mg, Cu 2.5 mg, Zn 50 mg, I 1.0 mg， Se 0.15 mg.

[b] Calculated value.

Table S2. The effect of DNJ extract from mulberry leaves on production performance in laying hens

Item	Supplementation DNJ-E levels (mg/kg)				S.E.M	*P-value*
	0	50	100	150		
Initial laying rate (%)	81.84	81.05	80.57	81.36	0.139	0.856
Initial body weight (g)	1.87	1.89	1.88	1.89	0.203	0.765
Laying rate (%)	81.76[b]	81.32[b]	79.32[b]	84.26[a]	0.011	0.043
Final laying rate (%)	82.44[a]	79.76[b]	81.54[ab]	82.14[a]	0.029	0.047
Final body weight (g)	1.91	1.89	1.90	1.91	0.213	0.812
Egg weight (g)	61.65	60.05	60.76	58.61	0.493	0.207
Average daily feed intake (g/d)	121.37	114.43	117.07	117.12	8.001	0.606
Feed-to-egg ratio	2.65	2.52	2.64	2.49	0.034	0.245

[a,b]Means in the same row without common superscripts differ significantly ($p < 0.05$).
